# Anti-Hypertensives Reduce the Rate of Alzheimer’s Disease Progression: A Cohort Study Linked with Genetic and Neuropathological Analyses

**DOI:** 10.14283/jpad.2024.156

**Published:** 2024-08-26

**Authors:** Zohara Sternberg, R. Podolsky, J. Yu, S. Hua, S. Halvorsen, D. Hojnacki, B. J. Schaller

**Affiliations:** 1 Department of Neurology, Stroke Center, Buffalo Medical Center, Buffalo, NY 14203 USA; 2 Department of Biostatistics, University at Buffalo, Buffalo, NY USA; 3 Department of Pharmacology and Toxicology, University at Buffalo, Buffalo, NY USA; 4 Institute of Physiopathology, Department of Pathology, University of Buenos Aires, Buenos Aires, Argentina

**Keywords:** Alzheimer’s disease, cognition, cohort study, CSF biomarkers, dementia, disease progression, hypertension, neuropathology

## Abstract

**Background:**

Arterial hypertension contributes to both the development and progression of dementia due to both Alzheimer’s disease (A.D.) and vascular pathology. However, the effects of different classes of anti-hypertensives (A.H.T.s), on the rate of dementia progression and brain neuropathology are unknown.

**Objective:**

To investigate the effect of each class of A.H.T., both as single and combined, on the rate of dementia progression. In addition, we analyzed the effect of A.H.T.s on brain neuropathology in AD participants, indicated by Braak staging, hippocampal atrophy, and baseline CSF levels of A-β42, total (T) tau, and P-181 tau.

**Methods:**

We have used the National Alzheimer’s Coordinating Center (NACC) Uniform Data Set (UDS).

**Results:**

A.H.T.s were associated with reduced yearly increase in the CDR-SOB scores of 1.025 during a 10-year follow-up (P<0.001). The overall survival rate was higher in A.H.T. users than non-users [HR: 0.912: 0.860, 0.967) P=0.002]. These trends continued when stratifying participants by age, gender, and APOE4 allele. Participants who did not use A.H.T.s had a mean yearly increase of 1.71±1.7 in the CDR-SOB scores. This value was reduced to 1.48±1.6, P=0.006 and 1.45±1.6, P=0.024 for participants with documented use of βB and A.R.B.s, respectively. Combining diuretics with α1-AB or ACEI led to synergistic effects in reducing the rise in CDR-SOB scores. The proportion of participants who were diagnosed having AD postmortem with severe Braak staging was significantly lower in A.H.T.-users than non-users. The severity of Braak staging, and hippocampal atrophy differed in participants >70 vs. <70 years old, in both males and females. A significant relationship was observed between hippocampal atrophy and Braak staging; and between hippocampal atrophy and baseline CSF levels of P-181 tau.

**Conclusion:**

Our results could have implications for halting the progression of dementia regardless of the etiology being related to AD or vascular pathology. The choice of combination of A.H.T. therapy should also consider the combination which would lead to an optimum benefit in slowing the progression of dementia. Additionally, our results underline a more complex A.D. disease model than previously thought, which opens new treatment options.

## Introduction

Alzheimer’s disease (A.D.) is one of the most common age-related neurodegenerative disorders and the most frequent cause of dementia involving a progressive cognitive decline, including memory, language, and executive function, accounting for up to 75% of all dementia. More than 4 million new cases are diagnosed each year with numbers expected to double by 2030 (1). A.D. is characterized by the abnormal aggregation and accumulation of beta-amyloid (Aβ) in the form of extracellular plaques and aggregation of hyper-phosphorylated tau protein in the form of intracellular neurofibrillary tangles (NFT) (2). The disease has a long asymptomatic phase followed by a symptomatic pre-dementia phase known as mild cognitive impairment (MCI) and a progressive dementia stage (3). Surrogates of cognitive functions such as neuropsychological testing, biomarkers, neuroimaging, or a combination of these measures are used to define the various stages of the disease (4).

MCI patients convert to clinically probable A.D. at a rate of 10–15% during follow-up, amounting to approximately 80% after a follow-up of 6 years (5). Progression to A.D. varies depending on several factors, including the presence of the APOE4 allele (6), and the degree of hippocampal atrophy (7), among others. The latter has been shown to correlate with cognitive function as assessed by the mini-mental state examination (MMSE) test scores (8). Longitudinal SPECT studies in A.D. patients have shown an inverse correlation between age and cognitive deterioration, assessed by the mean annual changes in the MMSE scores (9), and a positive correlation between A.D. progression and whole brain atrophy (10, 11). A.D. progression is also based on Braak staging of A.D. tauopathy, and a relationship between baseline tau-PET and subsequent atrophy has been observed, particularly in younger patients (12).

Chronic hypertension is associated with cognitive impairment and dementia related to both A.D. and vascular pathology (13). Elevated arterial BP has been shown to accelerate the rate of cognitive deterioration in MCI patients (14). Furthermore, associations have been reported between hypertension and hippocampal atrophy (15) and tau pathology (16).

Besides these clinical data, little is known about their influence and linkage to surrogate features like neuroradiology, neuropathology, neurogenetics and neuroimmunology. But this knowledge is essential for implementing treatment changes or new treatment recommendations in the different stages of dementia related either to AD or vascular pathology. Therefore, the present study aims to link proven risk factors as seen in our previous work (17) with other surrogate biomarkers to further explore potential clinical endpoints and to investigate the influence of antihypertensive (A.H.T.) medications use on the rate of dementia progression, taking into consideration both AD and vascular dementia. The data are analyzed for the progression and death among participants with CDRSOB of =>3, stratified by age, gender, and the presence of APOE4 allele, during a 10 year follow-up. In addition, we analyzed the correlation between A.H.T. use and the available brain neuropathology assessed by immunohistochemistry and neuroimaging data, as well as the cerebrospinal fluid (CSF) biomarkers such as A-β42, T-tau and p-181tau, to gain further insights in participants who were diagnosed with AD upon post-mortem autopsy.

## Methods

The study was approved by the Internal Review Board of the State University of New York at Buffalo, Buffalo, NY, U.S.A., and all participants’ information were de-identified in the data set received from the National ‘Alzheimer’s Coordinating Center (NACC). Written informed consent is obtained from all participants and co-participants.

### Definition of Dementia for this Study

The diagnosis of dementia was considered CDR-SOB >=3. The definite diagnosis of AD is assumed to be by autopsy. These participants have a diagnosis of “Probable AD, which can be “pure AD” or “mixed AD” The latter can be a combination of AD and vascular dementia.

Further premises of the diagnoses are mentioned below in (a) further diagnostic, (b) psychiatric assessment, and (c) neurological examination as well as in the inclusion/exclusion criteria.

### Study Design

All data were obtained only from and validated by the NACC. From September 2005 to the specific data freeze of March 2020 (containing data on to Feb 2020) for Alzheimer’s Disease Research Centers (ADRCs) across the U.S.A. have been contributing data to the UDS, using a prospective, standardized, and longitudinal clinical evaluation of the subjects in the National Institute on Aging’s (N.I.A.’s) ADRC program. In each subject’s approximate annual UDS visit, the clinician completes data collection forms, covering topics from subject demographics to neurological examination findings, neuropsychological test results and psychiatric symptoms or other diagnoses issues on individuals with normal cognition, mild cognitive impairment, and dementia. For each ADRC visit, a multidisciplinary team or a single clinician determines a clinical diagnosis based on established (national) guidelines.

### Study Population

The UDS reflects the total enrollment of the N.I.A.’s ADRCs from 2005 up to the data freeze of March 2020. Each Center enrolls its participants according to its protocol — e.g., clinician referral, self-referral by participants or family members, active recruitment in the community organizations, etc. Most centers also enroll volunteers with normal cognition and highly educated. Overall, participants are enrolled using different methods and for different research purposes at the N.I.A.’s ADRCs.

In this large prospective, standardized clinical case series rather than a longitudinal study in a strict sense, the analysis is based on complete covariates cases assuming that the missing patterns in covariates are random and do not depend on observed or unobserved observations. Loss of follow-up is right-censored data, incorporated in the data analysis.

### Inclusion/Exclusion Criteria

Inclusion criteria: (i) Participants with CDR-SOB>=3.

Exclusion criteria: (i) Participants 90 years or older; (ii) Participants with no or incomplete records of A.H.T. use for analysis; (iii) participants who died at their first record of CDR-SOB, with no follow-up.

Participants who took any A.T.H. at least once were compared to those who never used A.H.T.s. The results were limited to a 10 year follow-up and corrected for mental disease signs indicated by the use of antipsychotic drugs, due to a relationship between psychiatric diseases and cognitive deficit (18), Parkinson’s disease (P.D.), history of traumatic brain injury (T.B.I.), alcohol abuse, active depression in the last two years, heart attack/cardiac arrest, education history, history of smoking, use of diabetes medications or lipid-lowering medications known to modulate cognitive function (19).

### PICO Framework

To ensure clinical evidence, a PICO framework (20) was applied with a Population consisting of an ADRC data pool. Interventions consisting of different A.H.T. treatments/prophylaxis focused primarily on A.D. progression. Comparisons between clinical and pathological A.D. progression in different subgroups and outcomes vary but are based on neuropsychological tests.

### Data Collection

For this study, the UDS data, from the whole ADRCs data pool are collected using different standardized evaluations of participants enrolled in ADRC clinics. Data are recorded directly on UDS forms (hard copy or electronic) during the evaluation process. Information is collected during in-person office visits, home visits, and telephone calls. In addition, Milestone Forms are used to document participant death and drop-out. The UDS is longitudinal, and its protocol requires an approximate annual follow-up for as long as the participant can be involved. Late-stage participants forced to drop out due to health may continue to be followed strictly for autopsy purposes. Trained clinicians and clinic personnel collect data from participants and their co-participants (usually a close friend or family member). Depending on a given and validated ADRC protocol, diagnosis is made by either the consensus of the involved team or clinicians.

Although the focus of the ADRCs is A.D., the Centers also collect data on various associated disorders, such as vascular dementia, Lewy body dementia, and frontotemporal lobar degeneration (FTLD). Furthermore, the use of medications — e.g., A.H.T.s, hypolipidemics, antidiabetics, antidepressants, and antipsychotics is documented at each visit and during follow-up. However, completing the form assessing the participant’s use of medications is optional; therefore, completing the record of adherence to the treatment regimen may be incomplete.

### Data Management

To ensure patient privacy, the stored and transmitted data are de-identified at the participants and organization level. Structured data recorded in the electronic health records are assimilated into the database need, not meeting the data to standard and controlled clinical terms. A rigorous data quality assessment excludes records that do not meet quality standards and basic formatting requirements for adequate data representation.

### Further Diagnostic Evaluation

For some UDS participants, CSF values are available for A-β42, T-tau and P-181 tau, and were used for the current study. In the presented data set here, genotypic data (i.e., APOE status) is available at NACC for 75% of UDS participants, as well as genetic information on whether the participant or their family has any known A.D. or FTLD mutations.

### Psychiatric Assessment

Psychiatric symptom data included (i) a history of depression (coded as consulting a clinician, (ii) being prescribed medication or receiving a diagnosis related to depressed mood), (iii) a depressive symptoms scale (Geriatric Depression Scale-Short Form), (iv) history of pseudobulbar affect, and (v) history of substance use disorder and the Neuropsychiatric Inventory Questionnaire (NPI-Q) assessing presence/absence of 12 neurobehavioral symptoms Validation of the NPI-Q, a brief clinical form of the Neuropsychiatric Inventory.

### Neurological Examination

For this study, we have used a cohort of participants from NACC-UDS, limiting it to participants who enrolled having Clinical Dementia Rating Scale Sum of Boxes (CDR-SOB) of =>3 as assessed by Dementia Staging Instrument, with a follow-up of 10 years during disease progression and possible death. In addition, we have provided the values for the group differences in CDR®, MMSE scores and Montreal cognitive assessment (MoCA) scores. We defined the following subgroup analyses to investigate heterogeneous results for the primary outcome based on different drug classes or combinations of different drug classes.

### Statistical Analysis

The S.A.S. (Version 9.4) and R 4.0.2 software were used for all statistical analyses. We used χ2 tests for categorical variables and independent two sample t-test for continuous variables for univariate data analysis. The survival outcome was defined as the time from progression of cognitive decline, characterized by an increase in the CDR-SOB scores to greater than 3 over a follow-up of 10 years. In case of missing baseline demographics data, the first non-missing value from the repeated measure data was used. We compared between A.H.T.-users (minimum of one-time use) and non-users, with further stratifications based on age, gender, and at least one copy of the APOE4 allele.

We employed mixed-effects model with an interaction between follow-up year and A.H.T drug treatment to evaluate the changes in CDR-SOB scores due to the A.H.T. regimen over time. We incorporated random intercept in the model to account for within-subject variability in baseline CDR-SOB scores among participants.

Additionally, we determined the proportion of participants’ survival who had baseline value of CDRSOB =>3 and died during the 10-year follow up. The survival curves were estimated using Kaplan-Meier (K.M.) curve and the log-rank test to assess the effectiveness of A.H.T. drugs. The unadjusted Cox proportional hazard model estimated the hazard ratios between A.H.T.-users and non-users. Further, we performed the adjusted survival analysis using the additive hazards regression model incorporating covariates with moderating effects on survival and determine the association between the covariates and the time-to-death outcome. Covariates include P.D. status, history of T.B.I., vitamin B12 deficiency, alcohol abuse, smoking, education, depression and the use of antipsychotic, hypolipidemic, and antidiabetic medications during the follow-up period. Participants lost to follow-up were considered right censored and were appropriately handled in the data analysis.

To investigate the potential benefits of combination drug therapy, we explored the effects of combining common A.H.T. drugs using CDR-SOB average changes per year in comparison to changes in CDR-SOB on a single A.H.T regimen. The CDR-SOB changes per year were calculated by subtracting the CDR-SOB at the progression of the disease from the CDR-SOB at the last follow-up, then dividing by the number of years of follow-up. To create the combination drug, we chose to explore a combination of A.H.T drugs, often used in clinical practice. We assigned a value 1 if the participant used both drugs on the same visit and compared each combination drug with a single drug for statistical significance. We compared the effect of combination drugs such as diuretics, vasodilators, or α1-adrenergic blockers (α1-AB) often prescribed as addition to the main A.H.T. such ad C.C.B.s, β-blockers (βBs), angiotensin-converting enzyme inhibitors (A.C.E.I.s) or angiotensin II receptor blockers (A.R.B.s). However, in some cases, the combinations of C.C.B.s +A.R.B.s and α1-AB+diuretics were also available for analysis. The data were corrected for all-interested covariates as described above.

Secondary objectives included the postmortem neuropathological findings by analyzing the severity of Braak staging (21), and the extent of hippocampal atrophy, as well as assessing the baseline levels of CSF biomarkers of A.D., such as A-β42, T-tau and p-181tau. Braak staging presents the gradual progression of NFT pathology from the transentorhinal region to the limbic system and ultimately the neocortex region (20). The association between the categorical data (Braak staging and severity of hippocampal atrophy) were tested using the χ2 tests. The differences in the CSF levels of the three A.D. biomarkers, A-β42, T-tau and p-181 tau, between A.H.T.-users and non-users were analyzed by student’s t-test as secondary outcomes. Kendall’s tau correlation analyzed the significance of the correlation between various variables of interest.

## Results

### Study Population

**Figure 1:**
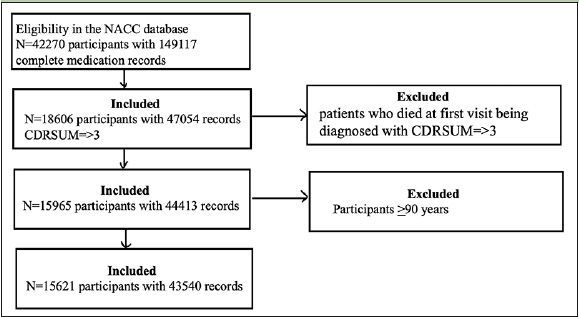
Flowchart of the study design

Initially, we had 42’270 participants with complete medication records. Among those, 18’606 participants had CDR-SOB of ≥3. We excluded participants who died at the first visit after being diagnosed with CDR-SOB >3 and remained with a sample size of 15’965. Next, we excluded participants who were ≥90 years old from this sample size, remaining with a sample size of 15’621 for analysis (Figure 1).

### Baseline Characteristics

Table 1A presents participants’ demographics. Among the 15’621 participants included in the analysis, the majority (61.7%) were, at least once, on one or more A.H.T.s. The average age of participants on A.H.T.s was significantly higher than participants who were not on A.H.T.s (74.2±8.7 vs. 69.4±10.5, P<0.001). Among male participants, 66.7% were receiving A.H.T drugs, whereas 57.3% of female participants were on A.H.T drugs (p<0.001). 38.5% of A.H.T.-users and 37.8% of nonusers were documented to have one or two APOE4 alleles (P=0.004). The median (136 mmHg (IQR: [123, 150]) vs. 130 mmHg (IQR: [120, 142])) and mean (137.09±19.8 mmHg vs. 131.56±17.8 mmHg) systolic arterial B.P. were significantly higher in A.H.T.-users vs. non-users (P<0.001). However, the median (75 mmHg (IQR: [69, 82]) vs. 75 mmHg (IQR: [69, 81]) and mean (75.27±10.9 mmHg vs. 74.98±10.2 mmHg) diastolic B.P. were not different between the two groups (P=0.127). Overall, the mean arterial B.P. (95.87±12.0 mmHg vs. 93.84±11.05 mmHg) was significantly higher in A.H.T.-users vs. non-users (p<0.001).

**Table 1A:** Participants’ characteristics. Abbreviations: W/AHT: use of antihypertensives; W/O AHT: without antihypertensives

	**Overall**			**Male**			**Female**		
**Pt’s demographics**	**W/AHT**	**W/O AHT**	**P-value**	**W/AHT**	**W/O AHT**	**P-Value**	**W/AHT**	**W/O AHT**	**P-Value**
N (% total)	9651 (61.7)	5970 (38.3)	N/A	4995 (51.7)	2497 (41.8)	N/A	4656 (48.3)	3473 (58.1)	N/A
Age (years, mean±SD)	74.2±8.7	69.3±10.5	<0.001	73.5±8.7	68.7±10.6	<0.001	75.0±8.7	69.8±10.4	<0.001
APOE4 (1copy)			0.004			0.04			0.124
Yes (% total)	3717 (38.5)	2257 (37.8)		1885 (50.7)	922 (40.8)		1832 (49.2)	1335 (59.1)	
No (% total)	3503 (36.2)	1897 (31.7)		1905 (54.3)	826 (43.5)		1598 (45.6)	1071 (56.4)	
Missing data	2431(25.1)	1816 (30.4)		1205.0	749.0		1226.0	1067.0	
Arterial BP (median)									
Systolic (mmHg)	136 [123,150]	130 [120,142]	<0.001	134 [122,148]	130 [120,140]	<0.001	138 [125,150]	130 [120,142]	<0.001
Diastolic (mmHg)	75 [68,82]	75 [69,81]	0.127	76 [69,82]	76 [70,82]	0.046	75 [68,82]	74 [68,80]	0.001
Mean (mmHg)	95 [88,103]	93 [87,101]	<0.001	95 [87,103]	94 [87,101]	<0.001	96 [88,104]	93 [86,101]	<0.001

Table 1B presents the baseline group differences in neuropsychological tests. The A.H.T.-users had significantly lower mean C.D.R. (0.99± 0.6 vs. 1.07±0.6, P<0.001) and CDR-SOB (5.84± 3.3 vs. 6.25±3.7, P<0.001); and higher MoCA (16.64± 6.0 vs. 15.68±6.3, P<0.001) compared to non-users, but the MMSE scores did not significantly differ between A.H.T.-users and non-users (P>0.05).

**Table 1B:** Neuropsychological test scores

	**W AHT**	**W/O AHT**	**P-value**
Neuropsychological Scores (mean±SD)			
CDR	0.99± 0.6	1.07±0.6	<0.001
CDR-SOB	5.84± 3.3	6.25±3.7	<0.001
MoCA (raw)	16.64± 6.0	15.68± 6.3	<0.001
MMSE	23.61± 13.2	23.73± 16.4	0.68

Abbreviations: CDR: Clinical dementia rating; MMSE: Mini mental state examination; MoCA: Montreal cognitive assessment; SOB: Sum of boxes

Table 1C presents group differences in several covariates. The percent of A.H.T.-users who had P.D. (1.65% vs. 1.09%), and used antipsychotics (4.36% vs. 3.04%), hypolipidemics (32.9% vs. 10.6%), acetylcholinesterase inhibitors (AchEIs) (37.0% vs. 23.4%), and tobacco (27.4% vs. 15%) was higher than non-users (all Ps <0.001). For some conditions, including T.B.I., heart attack/cardiac arrest, and use of alcohol, the database differentiated between active and inactive state, the former referring to conditions occurring within a year of the UDS visit. As such, the percentage of A.H.T.-users with heart attack/cardiac arrest (4.51% vs. 0.57%, P<0.001) and alcohol use (3.81% vs. 2.09%, P=0.001) were higher compared to non-users in an inactive state, but these percentages did not differ among the two groups in the active state.

**Table 1C:** Comparison of covariates between AHT-users and non-users and the associated P values

**Covariables**	**W AHT**	**W/O AHT**	**P (univariate model)**
N			
PD (%)	236 (1.6)	156 (1.0)	<0.001*
TBI (%)			
Active	37 (1.6)	33 (1.4)	0.054
Inactive	162 (7.2)	173 (7.7)	0.337
Alcohol consumption (%)			
Active	120 (0.8)	67 (0.4)	0.897
Inactive	544 (3.8)	299 (2.1)	0.001
Depression (past 2 years) (%)	3798 (26.8)	2370 (16.7)	0.658
Antipsychotics (%)	679 (4.3)	473 (3.0)	<0.001*
Education (%)	6166 (39.8)	4193 (27.1)	0.808
Heart attack/cardiac arrest (%)			
Active	120 (0.8)	17 (0.1)	0.427
Inactive	645 (4.5)	81 (0.5)	<0.001
Tobacco use (%)	3802 (27.4)	2081 (15.0)	0.002*
Hypolipedemics (%)	5123 (32.9)	1650 (10.6)	0.0001*
Antidiabetics (%)	1405 (9.0)	216 (1.4)	0.98
AchEI	5769 (37.0)	3646 (23.4)	<0.001*

Abbreviations: AchEI: Acetylcholinesterase inhibitors; BP: Blood pressure; PD: Parkinson’s disease; TBI: traumatic brain injury.

### A.H.T.s and Disease Progression

**Figure 2:**
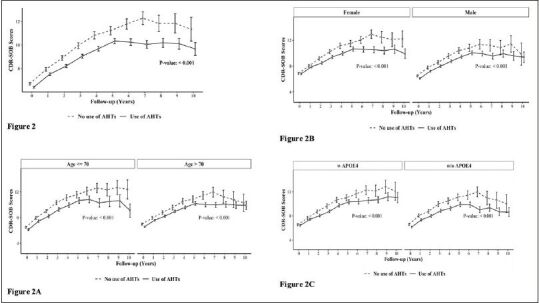
The effect of A.H.T.s on CDR-SOB scores during a 10-year follow-up; (Fig 2) as a function of age (≤70 vs. >70 years), (Fig 2A), gender (Fig 2B), and either one or two copies of the APOE4 allele (Fig 2C) Abbreviation: AHT: anti-hypertensive

Figure 2 demonstrates group differences in the rate of disease progression, indicated by increases in the CDR-SOB scores during the 10-year follow-up. A.H.T.-users (n=15621) had lower CDR-SOB by 1.025 (CI:0.878, 1.172) within the same follow-up year compared to nonusers (P<0.001). This trend continued when stratifying participants by age to <70 years (n=6037) [0.999 (0.774, 1.225), P<0.001] and >70 years (n=9584) [1.036 (0.835, 1.237) (P<0.001)] subgroups (Fig 2A); stratifying subjects based on female (n=8129) [0.994 [0.786, 1.202), p<0.001] and male (n=7492) [0.984 [0.775, 1.192), p<0.001] genders (Fig 2B), and the presence (n=5974) [0.926 (0.685, 1.166) (p<0.001)] or absence (n=5400) [1.351 (1.098, 1.604) (p<0.001)] of APOE4 allele (Fig 2C).

### A.H.T.s and Survival

**Figure 3:**
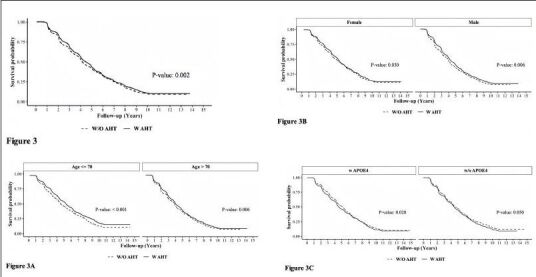
Adjusted survival curves using the Cox Proportional Hazards Model determining the effect of A.H.T.s on survival probability (Fig 3) during 10-year follow-up, as a function of age (≤70 vs. >70 years) (3A), gender (male/female) (3 B), and either one or two copies of APOE4 allele (3C). The K.M. curves were corrected for covariates, including Parkinson’s disease (P.D.), history of traumatic brain injury (T.B.I.), Vitamin B12 deficiency, alcohol abuse, education, smoking, cardiovascular disease, and reported use of antipsychotic agents

Figure 3 presents the survival curve estimates (KaplanMeier curves) for A.H.T. users and non-users under various stratifications. In the following, the unadjusted hazard ratio (HR) derived from the Cox model corresponding to the survival curves were presented. The overall rate of survival was higher among A.H.T.-users (n=15621) compared to non-users [HR: 0.912 (0.860, 0.967), p=0.002)] (Fig 3). This trend continued when stratifying participants by age to <70 years (n=6037) (HR: 0.812 [0.738, 0.894], p<0.001) and >70 years (n=9584) (HR: 0.896 [0.829, 0.968], p=0.006) subgroups (Fig 3A); by a female (n=8129) (HR: 0.908 [0.834, 0.989], p=0.030) and male (n=7492) (HR: 0.870 [0.802, 0.943], p<0.001) genders (Fig 3B), and by the presence (n=5974) (HR: 0.900 [0.825, 0.982], p=0.020) or absence (n=5400) (HR: 0.911 [0.831, 0.999], p=0.050) of APOE4 allele (Fig 3C).

Additive hazards regression model estimated the hazard differences between A.H.T.-users and nonusers, while adjusting for potential confounders such as the use of antipsychotic, antidiabetic, memantine, acetylcholinesterase inhibitors, history of heart attack, depression, traumatic brain injury, and arterial blood pressure, among other variables. Covariates with significant effects (p<0.001) were included in the final model, among these were the use of hypolipidemic medications, P.D. status, and baseline CDR-SOB scores. The results indicated that non-A.H.T.-users had an additional hazard of 0.031 (0.021, 0.041) compared to A.H.T.-users (p<0.001), suggesting that on average, there were 31 fewer deaths per 1,000 A.H.T.-users per year compared to non-users. This trend persisted when participants were stratified by age, sex, and the presence of APOE4 allele.

### A.H.T.s Drug and Combination Analysis

Table 2 presents the mean yearly increase (and the associated standard error) in CDR-SOB for the use of single (Table 2A) and combination (Table 2B) A.H.T.s. The analysis compares the benefit of each A.H.T. vs. the no use of A.H.T. Participants who did not use A.H.T.s had a mean yearly increase of 1.71±1.7 in the CDR-SOB scores. This value was reduced to 1.48±1.6, P=0.006 and 1.45±1.6, P=0.024 for participants with documented use of βB and A.R.B., respectively. Vasodilators (n=28) also decreased the mean CDR-SOB to 1.37±1.1, but without statistical significance (P=0.43). In addition, other A.H.T.s such as C.C.B., ACEI, α1-AB, and diuretics also reduced CDR-SOB scores, but the effect of these A.H.T.s also remained statistically insignificant (Table 2A).

**Table 2:** The effect of different classes of A.H.T.s as single (A) and in combination (B) on the annual rate of disease progression

**AHTs**	**N**	**Annual changes in CDR-SOB (Mean±SE)**	**P-value**
2A. Single AHT			
No AHT	4178	1.71±1.7	
VASD	28	1.37±1.1	0.429
ARB	333	1.45±1.6	0.024
βB	709	1.48±1.6	0.006
CCB	410	1.57±1.7	0.458
ACEI	695	1.59±1.7	0.254
DIUR	326	1.59±1.6	0.586
α1-AB	502	1.64±1.8	0.759
2B. Combination AHT			
α1-AB+DIUR	54	1.15±1.2	0.008 (vs. α1-AB)
ACEI+DIUR	263	1.22±1.4	0.000 (vs. ACEI)
ARB+DIUR	118	1.33±1.2	0.402 (vs. ARB)
βB +DIUR	206	1.46±1.7	0.876 (vs. βB)
βB + α1-AB	143	1.46±1.6	0.883 (vs. βB)
ARB+ CCB	124	1.46±1.5	0.930 (vs. ARB)
βB + VASD	37	1.47±1.9	0.985 (vs. βB)
CCB + ACEI	214	1.60 ±1.9	0.995 (vs. CCB)

Abbreviations: α1-AB: α1-adrenoceptor blocker; ACEI: angiotensin converting enzyme inhibitor; AHT: antihypertensive; ARB: angiotensin II receptor blocker; βB: beta blocker; CCB: calcium channel blocker; DIUR: diuretic; VASD: vasodilator

Analysis of combination A.H.T.s showed a synergistic effect of combining diuretics with α1-A.B. (1.15±1.2, P=0.008) and diuretics with A.C.E.I (1.22±1.4, P=0.000) in reducing the rise in CDR-SOB scores, respectively compared to a single drug regimen. Other combinations also reduced the increase in CDR-SOB compared to a single drug but with no statistical significance (Table 2B).

### A.H.T.s and Post-Mortem AD Brain Neuropathology

We compared the severity of brain neuropathology between A.H.T.-users and non-users by analyzing the group differences in Braak staging, and hippocampal atrophy. Among 3229 participants (1378 females, 42.6%) with available postmortem autopsy data (mean age at death 73.0±10.1), 2060 were A.H.T.-users and 1169 were non-users. Regardless of the use of A.H.T.s, most participants demonstrated stage VI Braak staging. However, a lower proportion of AHT-users (42%) had stage VI Braak staging compared to the proportion of non-users (51%) (P=0.000), but the effect of A.H.T.s was not observed in lower Braak staging scores (I to V) (Figure 4A). The group differences between AHT-users and nonusers were more pronounced, limiting the Braak staging data analysis to 184 A.H.T.-users and 62 non-users for whom the primary pathology was documented be A.D. This analysis showed that 59% of AHT-users and 77.4% of non-users had Braak staging with V-VI severity (P=0.000) (results are not graphed). Subsequently, we conducted a sensitivity analysis comparing Braak staging I to VI in A.H.T.-users females and males stratified by age (<70 years vs. >70 years). The patterns of Braak staging in A.H.T.-users differed in participants ≥70 compared to <70 years participants, in both females (P=0.000) (Fig 4B) and males (P=0.007) (Fig 4C)

**Figure 4A:**
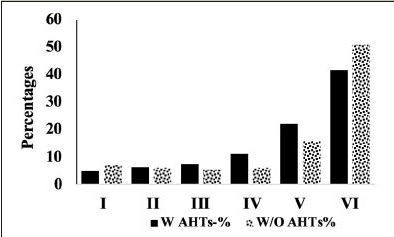
Braak staging: Comparison of proportion of A.H.T.-users and non-users at each Braak stage severity. The proportion of patrticipant at stage VI Braak stage severity was significanltly lower than A.H.T. non-users (42% vs. 51%, P=0.000)

**Figure 4B:**
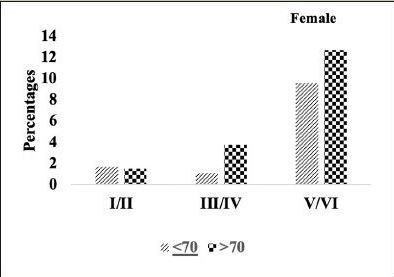
Braak staging: Differences in Braak staging in female A.H.T.-users stratified by age (P=0.000)

**Figure 4C:**
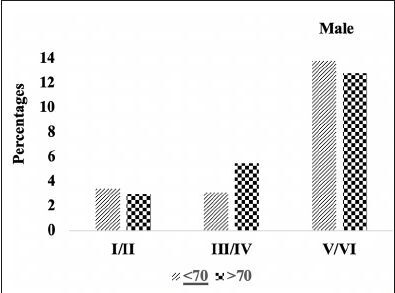
Braak staging: Differences in Braak staging in male A.H.T.-users stratified by age (P=0.007)

Comparison of stages of hippocampal atrophy (mild, moderate, severe) showed similar proportion of A.H.T.-users and non-users having severe hippocampal atrophy (13.9% vs. 14.8%) (P>0.05) (Figure 5A). However, when the analysis was limited to 74 A.H.T.-users and 23 nonusers in whom the primary pathology was documented to be A.D., 28.3% (n=21) A.H.T.-users and 34.8% (n=8) nonusers had severe hippocampal atrophy (P>0.05) (results are not graphed).

**Figure 5A:**
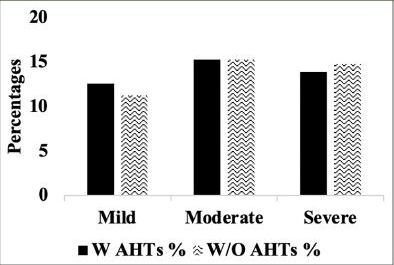
Hippocampal atrophy: Percentages of A.H.T.-users and non-users with mild, moderate and severe hipocampal atrophy. P>0.05.

**Figure 5B:**
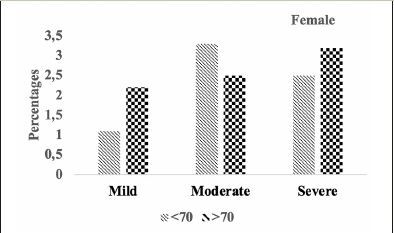
Hipocampal atrophy: Differences in hipocampal atrophy in female A.H.T.-users stratified by age (P=0.016)

**Figure 5C:**
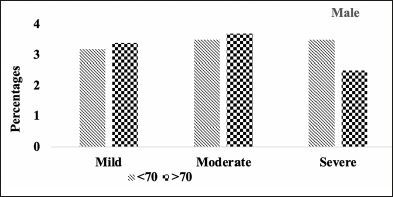
Hipocampal atrophy: Differences in hipocampal atrophy in male A.H.T.-users stratified by age (P=0.217)

Subsequently, we conducted a sensitivity analysis comparing the severity of hippocampal atrophy in A.H.T.- users females and males stratified by age (<70 years vs. >70 years). The patterns of hippocampal atrophy in A.H.T.-users differed in participants ≥70 compared to <70 years participants, in females (P=0.016) (Fig 5B), but not in males (P=0.217) (Fig 5C).

### A.T.H.s and CSF Biomarkers

We measured baseline CSF levels of A-β42, T-tau, and P-181 tau between A.H.T.-users and non-users. The baseline levels were analyzed since a 10-year follow-up values were unavailable. Although several methods in the NACC’s dataset measured these variables, we limited the analysis to baseline values assessed by the method of ELISA due to a higher sensitivity of this method over other measurement methods and better comparison. The data of 96 A.H.T.-users (71.4±7.4 years old, 34 females) and 70 non-users (68.1±10.4 years old, 36 females) could be analyzed for A-β42, and the data of 69 A.H.T.-users (70.8±8.0 years old, 25 females) and 46 nonusers (67.7±10.6 years old, 21 females) could be analyzed for T-tau and P-181 tau. The differences in A-β42 levels between A.H.T-users and non-users (386.3±177.2 pg/ml vs. 352.8±197.0 pg/ml) were not statistically significant (P=0.26). Similarly, the difference between A.H.T.-users and non-users in T-tau (697.1±396.6 pg/ml vs. 708.7±381.2 pg/ml, P=0.874) and P-181 tau (95.8±60.4 pg/ml vs. 98.7±56.1 pg/ml, P=0.79) remained insignificant. Similar patterns emerged when stratifying participants based on age (<70 years and >70 years).

### Correlation Studies

We subsequently correlated between Braak staging and hippocampal atrophy in A.H.T.-users and non-users, observing significant correlations between the two disease indicators, both in A.H.T.-users (coefficient = 0.2177, p<0.001) and non-users (coefficient= 0.1490, p<0.001). The correlation between baseline T-tau, p-181 tau and hippocampal atrophy showed a significant correlation between tau and hippocampal atrophy in A.H.T.-users (p-181 tau: P=0.007; T-tau: P=0.052) but not in non-users (p-181 tau: P=0.382; T-tau: P=0.677). However, we did not observe significant correlations between baseline T-tau, p-181 tau and CDR-SOB.

## Discussion

We have conducted an exhaustive analysis of NACC participants’ dataset showing that some A.H.T. drugs, used as single or in certain combinations, have the potential to reduce the rate of A.D. progression and increase the rate of survival as demonstrated during a 10-year follow-up; significant effects that were independent of age, gender, and the presence of APOE4 allele, as well as independent of arterial B.P. We could show this effect on the meso-, makro- and molecular level. Although AD is the focus of this article, participants with no autopsy may have AD, vascular dementia or a mix of the two, unless post-mortem autopsy proves otherwise. Therefore, our results on the benefits of A.H.T. on reducing the progression of dementia and increasing survival rate apply to both AD dementia and dementia related to vascular pathology.

We have recently reported (17) the beneficial effects of A.H.T.s in delaying the normal to MCI conversion rate in elderly subjects, an effect modulated by age, gender, and APOE4 allele. This previous observation is unlike the current one where A.H.T.s beneficial effect in slowing A.D. disease progression was independent of age, gender, and APOE4 allele, suggesting that the three factors may modulate the initiation of the AD pathological cascade, but not the progression, giving new interventions possibilities for treatment.

Among A.H.T. agents, βB significantly reduced the rate of disease progression. Our results agree with Rosenberg et al. (22) reporting beneficial effects of βB in slowing the annual increase in CDR-SOB in A.D. participants who were followed for three years. A recent nation-wide retrospective cohort study of Danish residents with hypertension reports (23) the beneficial effects of βB to be closely associated with their ability to cross the B.B.B. The βB that cross the B.B.B. are more effective in preventive cognitive decline than those that cross the BBB poorly. It seems therefore that βB have not only a remodeling effect in the heart, but also in the brain where earlier effects could be found in a metanalysis of 2005 traumatic brain injury cases (24). But in our study, effectiveness on subgroups, dose-response, length of therapy, functional outcome, and quality of life after βB use are not yet explored fully.

One of the mechanisms accounting for the protective effects of the centrally acting βB in A.D. is their ability to reduce A-β, in part, by increasing the level of A-β degrading enzymes, such as insulin degrading enzyme (25). Another mechanism contributing to the beneficial effects of βB is to increasing CSF-dependent brain clearance of neurotoxic metabolites including A-β and tau (26). This effect is achieved by inhibiting brain norepinephrine known to regulate astrocyte cell volume, thereby reducing resistance to flow and increasing the clearance of waste products from the brain’s interstitium into the periphery (27). It remains unclear how long this effect lasts. Although in the heart this effect seems to be limited to approximately one year.

Several studies have reported the beneficial effects of A.R.B.s in delaying the progression of A.D. (28, 29). One mechanism for A.R.B.s reduction in disease progression may be attributed to lowering of A-β oligomerization (30). An additional mechanism of A.R.B. in A.D. is related to A.R.B.s’ modulation of R.A.S. involved in hypertension and cognitive dysfunction (31). However, A.C.E.I.s also downregulate R.A.S. (32). Still, our results and those of others (33) and that of a meta-analysis (34) show a lesser effect of A.C.E.I.s compared to A.R.B.s in reducing disease progression.

R.A.S. in the C.N.S. operates independently of R.A.S. in the periphery. Unlike the peripheral R.A.S., the C.N.S. R.AS. is thought to be primarily involved in functions critical to cognition, including learning and memory (35). The potential of each type of A.R.B. and A.C.E.I. to improve cognitive function may be closely associated with the ability of these compounds to cross the B.B.B. (36, 37). Therefore, the differential effects of A.R.B.s and A.C.E.I.s in reducing the CDR-SOB increase suggest a higher use of brain-penetrating A.R.B.s than brain-penetrating A.C.E.I.s by the ADRC participants. Another factor that could modulate the efficacy of A.C.E.I.s in reducing A.D. disease progression is related to the presence or absence of APOE4 allele (38).

Combining A.C.E.I.s and diuretics led to synergistic effects in reducing the increase in CDR-SOB. Diuretics, such as furosemide, are known to cross the B.B.B. (39), and have been shown to reduce A-β oligomers in the brain (30). Diuretic reduction of A-β oligomers partly explains the significant effect of this class of A.H.T.s in interfering with the increase in CDR-SOB when added to A.C.E.I. and α1-A.B. However, neither A.C.EI.s nor diuretics or α1-AB significantly affected CDR-SOB increase as a single A.H.T. regimen. In a rodent model of A.D., the α1-AB, prazosin, improves cognitive performance due to affecting the level of inflammatory markers (40), suggesting that a combination of α1-AB anti-inflammatory effects and diuretic reduction of brain A-β oligomers (30) may be required to exert a synergistic effect, reducing the rate of A.D. progression. However, we recommend further high-quality trials to better explore the mechanisms of action, effectiveness on subgroups, dose-response, length of therapy, functional outcome, and quality of life.

Notably, the patterns of clinical efficacy among AHTs differ when analysis is targeted to the conversion from normal to MCI (17) versus the A.H.T. beneficial effects in reducing the rate of A.D. disease progression. One A.H.T. that especially stands out is the class of C.C.B.s showing significant efficacy in reducing the rate of normal to MCI conversion both in our study (17) and that of others (41) when used both as a single regime and combined with other A.H.T. (17). However, this beneficial effect of C.C.B. is absent when analyzing the impact of A.H.T. on the rate of A.D. disease progression, suggesting that C.C.B. beneficial effects may be related to the prevention of inflammation and oxidative stress (42), and dysregulation of intracellular calcium (43), pathological processes marking the early stages of cognitive impairment. These results observed both in early-stage cognitive impairment and in progressive dementia argue against “one size fit all” A.D. disease model; and that the extent of the cognitive impairment should be among factors for choosing a suboptimal A.H.T. regimen.

The analysis of brain neuropathology in A.H.T.-users, compared to non-users, showed a lower proportion of A.H.T.-users having Braak staging and hippocampal atrophy in higher severity orders than non-users. The beneficial effect of A.H.T.s was more pronounced in participants for whom AD was the main contributor to the observed neuropathology, and the effect was modulated by age, suggesting that A.H.T.s may target specifically the pathology related to A.D., in an age-dependent manner. Furthermore, the beneficial effects of A.H.T.s in the severe stage of Braak staging suggest that A.H.T.s effects in A.D. neuropathology may be complex depending on the A.H.T.s’ class and that more than one mechanism may contribute to the spread of NFT pathology in various brain regions. This conclusion is supported by a study involving participants in the NACC database, showing that participants on A.R.B.s have less NFT neuropathology than participants on other A.H.T.s (44).

The observed correlation between Braak staging and hippocampal atrophy further suggests the interrelationship between the two and that the presence of tau pathology may promote hippocampal atrophy (45). This observation was further corroborated by the observed association between P-181 tau and hippocampal atrophy and a near significant correlation between T-tau and hippocampal atrophy in A.H.T. users, and agrees with results of earlier studies reporting a correlation between T-tau and P-181 tau; and hippocampal atrophy (46, 47). Furthermore, the observed association between tau and hippocampal atrophy in A.H.T.-users, unlike nonusers, suggests that A.H.T.s may modulate tau pathology and hippocampal atrophy via similar mechanisms; partly explaining the recent meta-analysis of more than 800 cases from Australia reporting benefit of A.H.T.’s treatment in late-mid and later life to prevent dementia (48).

Our results of the current study and our former work (17) challenges the current concepts of A.D. as a linear cascade of (irreversible) events (49) as mentioned at different points in this discussion. However, our study does not explain the details of a new A.D. disease concept as we have only examined the effect of A.H.T. on disease prevention/progression. But showing different disease behavior between prevention and progression (e.g. related to age, gender and APOE4 allele) speaks – at least – for different intervention points for prevention and progression what goes not in line with current A.D. disease concepts. That is so far important as it opens new treatment/prevention possibilities but is also another strong hint toward the hypothesis that the pathological state of A.D. is thus a system of positive feedback loops, leading to amplification of the initial perturbation, rather than a linear cascade as already mentioned earlier by others (49). Drugs, like A.H.T.s examined in the current study. may therefore be effective by targeting numerous points within the loops, rather than concentrating on upstream processes.

### Study limitations

Although the results of this study have significant implications in the treatment of older hypertensive patients with dementia, one should point to the limitations of the study, one of which is the incomplete record of the use of different medications, and the recorded history of participants’ compliance with various drugs, including the use of A.H.T. regimen; this is because completing the form assessing participant’s medications is optional. We have tried to overcome this limitation with strict inclusion criteria and sensitivity analyses. Additionally, there could be a selection bias because of lack of randomization within our specific study design, which again we have tried to overcome with the large study population and with strict inclusion criteria.

Confounding by indication significantly threatens the validity of non-experimental studies assessing medical interventions. The prescriber plays in such a design a central role (50). We set strong inclusion/exclusion criteria in the study design to avoid further confounding by indication and used different antihypertensive drugs to treat the same diagnosis. However, by using standardized protocols for the study design and data collection and treatment guidelines, we achieved a prescriber stratification that is considered the strongest bias reduction

Furthermore, it should be noted that the results observed for the group differences in the three CSF biomarkers cannot be conclusive since the standards of ELISA tests and the cutoff values for A-β, T-tau and P-181 tau varied among laboratories. But the large study population has partially overcome this limitation.

## Conclusion

On a large and representative sample size and with strict inclusion criteria, we could confirm that A.H.T. treatments benefit cognitive function, either alone or in specific combinations. This observation could be established on several levels: 1) psychological testing, 2) pathology/histology and 3) molecular level. The results have, therefore, direct clinical implications in treating elderly patients with cognitive dysfunction and the search for A.H.T.s drug combinations with the potential to halt the progression of the disease.

Based on our study, we recommend using A.H.T. treatment in late-mid and older patients. However, we recommend further high-quality trials to answer questions about the mechanisms of action, effectiveness on subgroups, dose-response, length of therapy, functional outcomes, and quality of life after A.H.T. use for A.D. progression, but also, on a more complex disease model.

## Data Availability

*Availability of data and materials:* Data and materials can be available upon request.

## Abbreviations

α1-AB: α1-adrenergic blockers
AD: Alzheimer disease
ADRC: Alzheimer’s Disease Research Centers
ACEI: Angiotensin-Converting Enzyme Inhibitors
A.R.B.: Angiotensin Receptor Blockers
βB: β-blockers
Aβ: β-amyloid
C.C.B.: Calcium Channel Blockers
CDR®: Dementia Staging Instrument
CDR-SOB: Clinical Dementia Rating Scale Sum of Boxes
MCI: Mild Cognitive Impairment
MMSE: Mini-Mental State Examination
MoCA: Montreal Cognitive Assessment
NACC: National Alzheimer’s Coordinating Center
N.I.A.: National Institute on Aging
NFT: Neurofibrillary Tangles
UDS: Uniform Data Set

## Notes

*Ethics Approval:* This is an observational study. The University at Buffalo Research Ethics Committee has confirmed that no ethical approval is required.

## Notes

*Consent to Participate:* NA.

## References

[1] Prince M, Bryce R, Albanese E, Wimo A, Ribeiro W, Ferri CP. The global prevalence of dementia: a systematic review and metaanalysis. Alzheimers Dement. 2013;9(1):63–75e2. 23305823 10.1016/j.jalz.2012.11.007

[2] Selkoe DJ. Alzheimer’s disease: genes, proteins, and therapy. Physiol Rev. 2001;81(2):741–66. 11274343 10.1152/physrev.2001.81.2.741

[3] Sperling RA, Aisen PS, Beckett LA, Bennett DA, Craft S, Fagan AM, et al. Toward defining the preclinical stages of Alzheimer’s disease: recommendations from the National Institute on Aging-Alzheimer’s Association workgroups on diagnostic guidelines for Alzheimer’s disease. Alzheimers Dement. 2011;7(3):280–92. 21514248 10.1016/j.jalz.2011.03.003 PMC3220946

[4] Vos SJ, Verhey F, Frolich L, Kornhuber J, Wiltfang J, Maier W, et al. Prevalence and prognosis of Alzheimer’s disease at the mild cognitive impairment stage. Brain. 2015;138 (Pt 5):1327–38. 25693589 10.1093/brain/awv029 PMC5013930

[5] Petersen RC. Mild cognitive impairment: transition between aging and Alzheimer’s disease. Neurologia. 2000;15(3):93–101. 10846869

[6] Petersen RC, Smith GE, Ivnik RJ, Tangalos EG, Schaid DJ, Thibodeau SN, et al. Apolipoprotein E status as a predictor of the development of Alzheimer’s disease in memory-impaired individuals. JAMA. 1995;273(16):1274–8. 7646655

[7] Jack CR, Jr., Petersen RC, Xu YC, O’Brien PC, Smith GE, Ivnik RJ, et al. Prediction of AD with MRI-based hippocampal volume in mild cognitive impairment. Neurology. 1999;52(7):1397–403. 10227624 10.1212/wnl.52.7.1397 PMC2730146

[8] Peng GP, Feng Z, He FP, Chen ZQ, Liu XY, Liu P, et al. Correlation of hippocampal volume and cognitive performances in patients with either mild cognitive impairment or Alzheimer’s disease. CNS Neurosci Ther. 2015;21(1):15–22. 25146658 10.1111/cns.12317 PMC6495306

[9] Sakai M, Hanyu H, Kume K, Sato T, Hirao K, Kanetaka H, et al. Rate of progression of Alzheimer’s disease in younger versus older patients: a longitudinal single photon emission computed tomography study. Geriatr Gerontol Int. 2013;13(3):555–62. 22963387 10.1111/j.1447-0594.2012.00934.x

[10] Spulber G, Niskanen E, MacDonald S, Smilovici O, Chen K, Reiman EM, et al. Whole brain atrophy rate predicts progression from MCI to Alzheimer’s disease. Neurobiol Aging. 2010;31(9):1601–5. 18829136 10.1016/j.neurobiolaging.2008.08.018

[11] Schaller BJ. Strategies for molecular imaging dementia and neurodegenerative diseases. Neuropsychiatr Dis Treat. 2008;4(3):585–612. 18830391 10.2147/ndt.s2154 PMC2526366

[12] La Joie R, Visani AV, Baker SL, Brown JA, Bourakova V, Cha J, et al. Prospective longitudinal atrophy in Alzheimer’s disease correlates with the intensity and topography of baseline tau-PET. Sci Transl Med. 2020;12(524). 10.1126/scitranslmed.aau5732 PMC7035952 31894103

[13] Wanleenuwat P, Iwanowski P, Kozubski W. Alzheimer’s dementia: pathogenesis and impact of cardiovascular risk factors on cognitive decline. Postgrad Med. 2019;131(7):415–22. 31424301 10.1080/00325481.2019.1657776

[14] Goldstein FC, Levey AI, Steenland NK. High blood pressure and cognitive decline in mild cognitive impairment. J Am Geriatr Soc. 2013;61(1):67–73. 23301925 10.1111/jgs.12067 PMC3699694

[15] Power MC, Schneider AL, Wruck L, Griswold M, Coker LH, Alonso A, et al. Life-course blood pressure in relation to brain volumes. Alzheimers Dement. 2016;12(8):890–9. 27139841 10.1016/j.jalz.2016.03.012 PMC4980244

[16] Arvanitakis Z, Capuano AW, Lamar M, Shah RC, Barnes LL, Bennett DA, et al. Late-life blood pressure association with cerebrovascular and Alzheimer disease pathology. Neurology. 2018;91(6):e517–e25. 29997190 10.1212/WNL.0000000000005951 PMC6105052

[17] Sternberg Z, Podolsky R, Yu J, Tian M, Hojnacki D, Schaller B. Delayed Decline of Cognitive Function by Antihypertensive Agents: A Cohort Study Linked with Genotype Data. J Prev Alzheimers Dis. 2022;9(4):679–91. 36281672 10.14283/jpad.2022.73

[18] Sheffield JM, Karcher NR, Barch DM. Cognitive Deficits in Psychotic Disorders: A Lifespan Perspective. Neuropsychol Rev. 2018;28(4):509–33. 30343458 10.1007/s11065-018-9388-2 PMC6475621

[19] Geifman N, Brinton RD, Kennedy RE, Schneider LS, Butte AJ. Evidence for benefit of statins to modify cognitive decline and risk in Alzheimer’s disease. Alzheimers Res Ther. 2017;9(1):10. 28212683 10.1186/s13195-017-0237-y PMC5316146

[20] Schardt C, Adams MB, Owens T, Keitz S, Fontelo P. Utilization of the PICO framework to improve searching PubMed for clinical questions. BMC Med Inform Decis Mak. 2007;7:16. 17573961 10.1186/1472-6947-7-16 PMC1904193

[21] Braak H, Braak E. Neuropathological stageing of Alzheimer-related changes. Acta Neuropathol. 1991;82(4):239–59. 1759558 10.1007/BF00308809

[22] Rosenberg PB, Mielke MM, Tschanz J, Cook L, Corcoran C, Hayden KM, et al. Effects of cardiovascular medications on rate of functional decline in Alzheimer disease. Am J Geriatr Psychiatry. 2008;16(11):883–92. 18978249 10.1097/JGP.0b013e318181276a PMC2676234

[23] Beaman EE, Bonde AN, Larsen SMU, Ozenne B, Lohela TJ, Nedergaard M, et al. Blood-brain barrier permeable beta-blockers linked to lower risk of Alzheimer’s disease in hypertension. Brain. 2023;146(3):1141–51. 35196379 10.1093/brain/awac076 PMC9976965

[24] Alali AS, Mukherjee K, McCredie VA, Golan E, Shah PS, Bardes JM, et al. Beta-blockers and Traumatic Brain Injury: A Systematic Review, Meta-analysis, and Eastern Association for the Surgery of Trauma Guideline. Ann Surg. 2017;266(6):952–61. 28525411 10.1097/SLA.0000000000002286 PMC5997270

[25] Dobarro M, Gerenu G, Ramirez MJ. Propranolol reduces cognitive deficits, amyloid and tau pathology in Alzheimer’s transgenic mice. Int J Neuropsychopharmacol. 2013;16(10):2245–57. 23768694 10.1017/S1461145713000631

[26] Xie L, Kang H, Xu Q, Chen MJ, Liao Y, Thiyagarajan M, et al. Sleep drives metabolite clearance from the adult brain. Science. 2013;342(6156):373–7. 24136970 10.1126/science.1241224 PMC3880190

[27] Sherpa AD AC, Hrabetova S. Noradrenaline drives structural changes in astrocytes and brain extracellular space. In: Vardjan N and Zorec R, eds. Noradrenergic Signaling and Astroglia. Elsvier. 2017;NA:241–55.

[28] Chiu WC, Ho WC, Lin MH, Lee HH, Yeh YC, Wang JD, et al. Angiotension receptor blockers reduce the risk of dementia. J Hypertens. 2014;32(4):938–47. 24406780 10.1097/HJH.0000000000000086

[29] Li NC, Lee A, Whitmer RA, Kivipelto M, Lawler E, Kazis LE, et al. Use of angiotensin receptor blockers and risk of dementia in a predominantly male population: prospective cohort analysis. BMJ. 2010;340:b5465. 20068258 10.1136/bmj.b5465 PMC2806632

[30] Zhao W, Wang J, Ho L, Ono K, Teplow DB, Pasinetti GM. Identification of antihypertensive drugs which inhibit amyloid-beta protein oligomerization. J Alzheimers Dis. 2009;16(1):49–57. 19158421 10.3233/JAD-2009-0925

[31] Royea J, Hamel E. Brain angiotensin II and angiotensin IV receptors as potential Alzheimer’s disease therapeutic targets. Geroscience. 2020;42(5):1237–56. 32700176 10.1007/s11357-020-00231-y PMC7525853

[32] Esteras R, Perez-Gomez MV, Rodriguez-Osorio L, Ortiz A, Fernandez-Fernandez B. Combination use of medicines from two classes of renin-angiotensin system blocking agents: risk of hyperkalemia, hypotension, and impaired renal function. Ther Adv Drug Saf. 2015;6(4):166–76. 26301070 10.1177/2042098615589905 PMC4530349

[33] Deng Z, Jiang J, Wang J, Pan D, Zhu Y, Li H, et al. Angiotensin Receptor Blockers Are Associated With a Lower Risk of Progression From Mild Cognitive Impairment to Dementia. Hypertension. 2022;79(10):2159–69. 35766029 10.1161/HYPERTENSIONAHA.122.19378

[34] Scotti L, Bassi L, Soranna D, Verde F, Silani V, Torsello A, et al. Association between renin-angiotensin-aldosterone system inhibitors and risk of dementia: A meta-analysis. Pharmacol Res. 2021;166:105515. 33636351 10.1016/j.phrs.2021.105515

[35] Llorens-Cortes C, Mendelsohn FA. Organisation and functional role of the brain angiotensin system. J Renin Angiotensin Aldosterone Syst. 2002;3 Suppl 1:S39–48. 12428219 10.3317/jraas.2002.029

[36] Sink KM, Leng X, Williamson J, Kritchevsky SB, Yaffe K, Kuller L, et al. Angiotensin-converting enzyme inhibitors and cognitive decline in older adults with hypertension: results from the Cardiovascular Health Study. Arch Intern Med. 2009;169(13):1195–202. 19597068 10.1001/archinternmed.2009.175 PMC2881686

[37] Ho JK, Nation DA, Alzheimer’s Disease Neuroimaging I. Memory is preserved in older adults taking AT1 receptor blockers. Alzheimers Res Ther. 2017;9(1):33. 28446207 10.1186/s13195-017-0255-9 PMC5405458

[38] Qiu WW, Lai A, Mon T, Mwamburi M, Taylor W, Rosenzweig J, et al. Angiotensin converting enzyme inhibitors and Alzheimer disease in the presence of the apolipoprotein E4 allele. Am J Geriatr Psychiatry. 2014;22(2):177–85. 23567418 10.1016/j.jagp.2012.08.017 PMC3873370

[39] Javaheri S, Corbett W, Adams JM, Davis PJ, Gartside PS. Acute respiratory acidosis: large-dose furosemide and cerebrospinal fluid ions. J Appl Physiol (1985). 1994;76(6):2651–5. 7928896 10.1152/jappl.1994.76.6.2651

[40] Katsouri L, Vizcaychipi MP, McArthur S, Harrison I, Suarez-Calvet M, Lleo A, et al. Prazosin, an alpha(1)-adrenoceptor antagonist, prevents memory deterioration in the APP23 transgenic mouse model of Alzheimer’s disease. Neurobiol Aging. 2013;34(4):1105–15. 23063647 10.1016/j.neurobiolaging.2012.09.010

[41] Forette F, Seux ML, Staessen JA, Thijs L, Babarskiene MR, Babeanu S, et al. The prevention of dementia with antihypertensive treatment: new evidence from the Systolic Hypertension in Europe (Syst-Eur) study. Arch Intern Med. 2002;162(18):2046–52. 12374512 10.1001/archinte.162.18.2046

[42] Chakroborty S, Stutzmann GE. Early calcium dysregulation in Alzheimer’s disease: setting the stage for synaptic dysfunction. Sci China Life Sci. 2011;54(8):752–62. 21786198 10.1007/s11427-011-4205-7

[43] Song T, Song X, Zhu C, Patrick R, Skurla M, Santangelo I, et al. Mitochondrial dysfunction, oxidative stress, neuroinflammation, and metabolic alterations in the progression of Alzheimer’s disease: A meta-analysis of in vivo magnetic resonance spectroscopy studies. Ageing Res Rev. 2021;72:101503. 34751136 10.1016/j.arr.2021.101503 PMC8662951

[44] Hajjar I, Brown L, Mack WJ, Chui H. Impact of Angiotensin receptor blockers on Alzheimer disease neuropathology in a large brain autopsy series. Arch Neurol. 2012;69(12):1632–8. 22964777 10.1001/archneurol.2012.1010 PMC3608189

[45] Josephs KA, Murray ME, Tosakulwong N, Whitwell JL, Knopman DS, Machulda MM, et al. Tau aggregation influences cognition and hippocampal atrophy in the absence of beta-amyloid: a clinico-imaging-pathological study of primary age-related tauopathy (PART). Acta Neuropathol. 2017;133(5):705–15. 28160067 10.1007/s00401-017-1681-2 PMC6091858

[46] Tarawneh R, Head D, Allison S, Buckles V, Fagan AM, Ladenson JH, et al. Cerebrospinal Fluid Markers of Neurodegeneration and Rates of Brain Atrophy in Early Alzheimer Disease. JAMA Neurol. 2015;72(6):656–65. 25867677 10.1001/jamaneurol.2015.0202 PMC4551490

[47] Henneman WJ, Vrenken H, Barnes J, Sluimer IC, Verwey NA, Blankenstein MA, et al. Baseline CSF p-tau levels independently predict progression of hippocampal atrophy in Alzheimer disease. Neurology. 2009;73(12):935–40. 19770469 10.1212/WNL.0b013e3181b879ac PMC2839552

[48] Peters R, Xu Y, Fitzgerald O, Aung HL, Beckett N, Bulpitt C, et al. Blood pressure lowering and prevention of dementia: an individual patient data meta-analysis. Eur Heart J. 2022;43(48):4980–90. 36282295 10.1093/eurheartj/ehac584

[49] Doig AJ. Positive Feedback Loops in Alzheimer’s Disease: The Alzheimer’s Feedback Hypothesis. J Alzheimers Dis. 2018;66(1):25–36. 30282364 10.3233/JAD-180583 PMC6484277

[50] Franklin JM, Schneeweiss S, Huybrechts KF, Glynn RJ. Evaluating possible confounding by prescriber in comparative effectiveness research. Epidemiology. 2015;26(2):238–41. 25643103 10.1097/EDE.0000000000000241 PMC4347927

